# Subacute Multivalvular Bacterial Endocarditis Complicated by Ruptured Mycotic Aneurysm and the Impact of Gender on Early Surgical Intervention

**DOI:** 10.7759/cureus.59771

**Published:** 2024-05-06

**Authors:** Michael Fragner, Sudarshan S Srivats, Jude Elsaygh, Kevin Pink

**Affiliations:** 1 Internal Medicine, New York-Presbyterian Brooklyn Methodist Hospital, Brooklyn, USA; 2 Internal Medicine, Catholic Medical Center, Manchester, USA; 3 Cardiology, New York-Presbyterian Brooklyn Methodist Hospital, Brooklyn, USA

**Keywords:** mycotic aneurysms, embolic events, infective endocarditis cerebral infarction, infective endocarditis complications, multi-valve endocarditis, subacute bacterial endocarditis, bivalvular endocarditis

## Abstract

Subacute bacterial endocarditis (SBE) evolves over weeks to months, often without typical features of acute endocarditis. Its presentation progresses gradually until possibly complicated by sentinel events, such as a cerebrovascular accident from embolization or a ruptured vessel. This is a case of SBE presenting as symptomatic anemia in a female patient with severe aortic regurgitation (AR) and mitral regurgitation (MR) due to bi-valvular vegetations in the absence of typical acute endocarditis and congestive heart failure (CHF) features.

## Introduction

In bacterial endocarditis, 90% of patients present with fevers, night sweats, fatigue, and weight loss, and 25% present with embolic sequelae [1]. Neurological sequelae are the most common extra-cardiac complications (25-70%), and mycotic aneurysms, or infectious intracranial aneurysms (IIA), are present in 2-4% of cases [2]. In one study, 80% mortality was found in IIA rupture versus 30% in unruptured cases, although unknown if due to re-bleeding or initial hemorrhage [2]. It is theorized that underlying bacteremia causes a pro-inflammatory state, influencing peripheral nerve function, leading to weakness and neuronal deterioration [3]. 

Multivalvular endocarditis is an uncommon, historically understudied, and clinically challenging diagnosis, seen in 12-30% of endocarditis cases [4]. Alvarez-Zaballos et al. retrospectively analyzed differences in patients with monovalvular and multivalvular endocarditis among 4,064 definitive infective endocarditis (IE) cases. They found a higher incidence of intracardiac complications (36.2% vs. 50.4%), heart failure (42.7% vs. 52.9%), and in-hospital mortality (26.9% vs. 34.3%) in the multivalvular cohort (14.2% of the cases) [4]. Bohbot et al. additionally determined a difference in multivalvular endocarditis compared to monovalvular regarding embolic events and 30-day mortality (34.5% vs. 17.6%) [5].

Our patient’s benign presentation did not correlate with the extent of her disease, leading to a delay in surgical intervention and severe neurological consequences. There are real differences in the management of IE between genders. Sambola et al. noted in their prospective observational cohort study that women have surgery performed less frequently (26% vs. 47%), and this results in worse outcomes compared to men (in-hospital mortality and one-year mortality) [6]. This case of severe valvular destruction in a relatively asymptomatic female patient highlights the importance of early surgical intervention, especially in women.

## Case presentation

**Figure 1:**
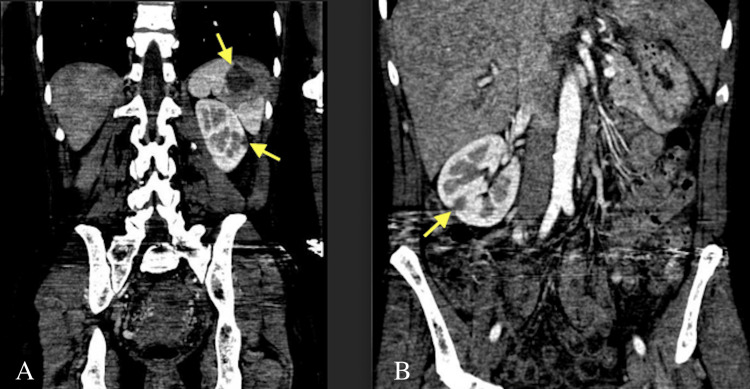
CT abdomen and pelvis coronal view performed on admission (A) Top arrow indicating hypodense splenic infarct, bottom arrow indicating hypodense left renal infarct. (B) Arrow indicating hypodense right renal infarct.

**Figure 2:**
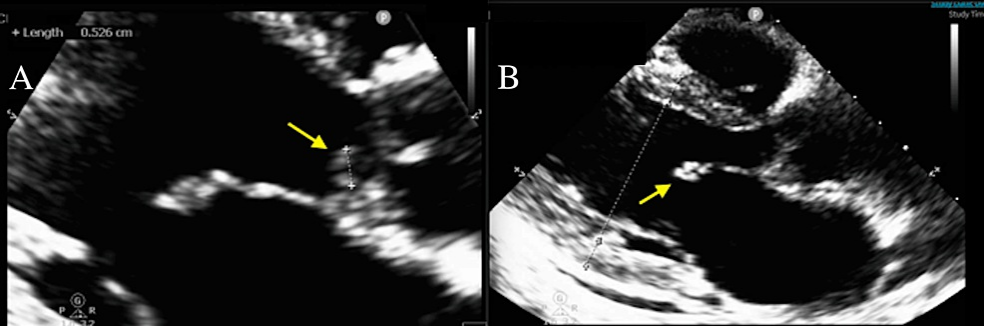
Transthoracic echocardiogram (TTE) long-axis view (A) Arrow indicating aortic valve vegetation. (B) Arrow indicating mitral valve vegetation.

A 53-year-old female with a history of right occipital 5 mm meningioma and resection via right frontotemporal craniotomy and left posterior communicating artery 2 mm brain aneurysm was sent to the emergency room by her primary medicine doctor for a decrease in hemoglobin to 7.8. She reported fatigue, arthralgias, headache, subjective fevers, palpitations, and weight loss for the past few months. Lab exams were notable for diastolic murmur III/VI best heard in the aortic zone and chronic right mid-face paresthesia that has been present since her previous brain surgery. Hemoglobin on presentation was 7.7, and she had mild leukocytosis. She was tachycardic, mildly hypotensive but responsive to intravenous fluids and Trendelenburg positioning, and febrile. There was concern for underlying abdominal malignancy, and CT abdomen and pelvis was performed showing splenic and bilateral renal infarcts (Figure 1). Transthoracic echocardiogram (TTE) showed an ejection fraction of 55% with vegetations on mitral and aortic valves with severe aortic regurgitation (AR) and mitral regurgitation (MR) (Figure 2).

**Figure 3:**
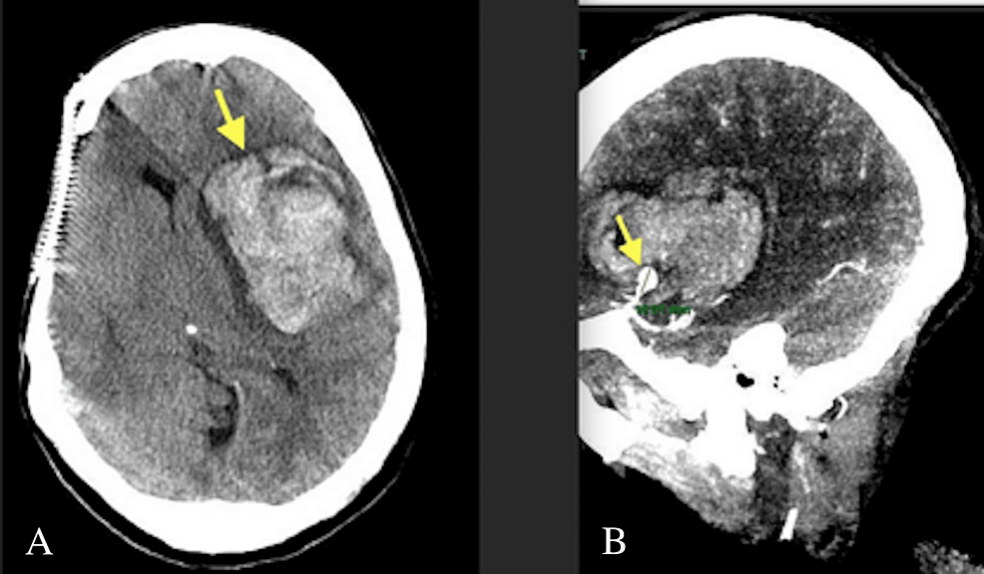
CT head performed after neurological deterioration (A) Arrow indicating intracerebral hemorrhage due to a ruptured mycotic aneurysm. (B) Arrow indicating increased 10 mm mycotic aneurysm (from baseline 3 mm, not shown) after rupture.

She was started on broad-spectrum antibiotics, and blood cultures grew *Streptococcus viridans*. On dental examination, tooth #5’s lingual cusp was broken, and there was pain on palpation in the apical area between teeth #4 and #5, but there was no sign of oral abscess or infection. Transesophageal echocardiogram (TEE) was unable to be performed due to anatomical difficulty, yet ENT cleared of any upper airway masses or obstructions. She was transferred to cardiothoracic surgery service to undergo evaluation prior to valve replacement. Pre-operative screening MRI brain for potential vegetation embolization showed a mycotic aneurysm and evolving subacute ischemic changes in the left middle cerebral artery. Later that same day, the patient developed an altered mental status and severe neurological deficits. CT head showed an acute embolic stroke, mycotic aneurysm expansion with hemorrhage, and midline shift due to aneurysm rupture (Figure 3). Her course included various neurosurgeries, intubation and tracheostomy placement, and worsening valvulopathy in the aortic and mitral valve. The patient became a poor surgical candidate, her endocarditis had to be treated solely medically, her mental status never returned to baseline, and her course unfortunately proved to be fatal months later.

## Discussion

In 30-50% of cases in IE, cardiac complications are present. However, our patient was asymptomatic and without signs of valvular disease or congestive heart failure despite extensive disease discovered on echocardiography [7]. Importantly, simultaneous native aortic and mitral valve infection has been correlated with poor prognosis (mortality rate ~56%) [3]. Multivalvular involvement is common in left-sided native valve IE and is associated with increased embolic events and a higher prevalence of CHF than singular valve involvement. Studies have shown a higher 30-day mortality rate in multivalvular IE (24.5%) versus monovalvular (17.6%), in addition to a lower 10-year survival (59% and 70%, respectively). Importantly, early surgery was associated with increased survival in multivalvular cases (79% vs. 35%) [5]. 

Hemodynamic status, however, is primarily what indicates the timeline and necessity of operation. According to the American College of Cardiology (ACC)/American Heart Association (AHA) task force on recommendations for surgery in IE, surgery is indicated in patients with IE who present with valvular destruction and heart failure symptoms. This is an emergency and must be corrected immediately during initial hospitalization and before completion of the course of antibiotics [8].

The two other main indications for surgery in native valve IE are uncontrolled infection and prevention of embolization (vegetation >10 mm with embolic events while on antibiotics or with severe valve disease/mobile vegetation, vegetation >30 mm) [1,9]. Relative indications, such as recurrent fevers while on antibiotics, multiple vegetations, and severe valvular disease, were all present in our patient and elevated her risk for embolization and neurological complications. Embolic events have been shown to occur at a 30-40% rate in left-sided definite IE with a known correlation of mortality, likely explained by the increased vegetation burden. Early valve surgery in these cases very well may decrease the incidence of embolism [10].

Sambola et al. were able to show that male gender was an independent factor affecting surgical treatment. This is an important point because surgery was associated with improved in-hospital and one-year mortality in both cohorts regardless of gender. Their results indicated that women underwent surgery less often, and mortality was higher overall long term [10]. This correlates with their findings, as the most frequent cause of death in the women cohort was congestive heart failure, a likely complication of IE linked to a delay in surgery [6].

## Conclusions

The most important surgical indication in endocarditis is valvular destruction with symptomatic disease, especially in cardiogenic shock. Acute aortic and mitral regurgitation is an emergency, it must be corrected immediately, and clinicians must continue to be objective, yet mindful, in women with IE who are candidates for early and aggressive surgical intervention. There appears to be a systemic bias in the acute management of women with surgically IE. Whether this is a systematic bias or a result of a more obscure presentation, there must be further studies examining if there truly is a difference in how we suspect endocarditis in females compared to males, in addition to differences in how we manage patients with curative surgery. The devastating nature of endocarditis cannot be taken lightly, and as clinicians, we must be objective, swift, and mindful of our biases when considering endocarditis in women with an ambiguous presentation and non-typical risk factors.
